# Prostate cancer screening research can benefit from network medicine: an emerging awareness

**DOI:** 10.1038/s41540-020-0133-0

**Published:** 2020-05-07

**Authors:** Valeria Panebianco, Martina Pecoraro, Giulia Fiscon, Paola Paci, Lorenzo Farina, Carlo Catalano

**Affiliations:** 1 Department of Radiological Sciences, Oncology and Pathology, Sapienza University/Policlinico Umberto I of Rome, Rome, Italy; 2 Institute for System Analysis and Computer Science (IASI), National Research Council, Rome, Italy; 3 Department of Computer, Control and Management Engineering, Sapienza University of Rome, Rome, Italy

**Keywords:** Molecular biology, Cancer, Computational biology and bioinformatics, Biomarkers, Urology

## Abstract

Up to date, screening for prostate cancer (PCa) remains one of the most appealing but also a very controversial topics in the urological community. PCa is the second most common cancer in men worldwide and it is universally acknowledged as a complex disease, with a multi-factorial etiology. The pathway of PCa diagnosis has changed dramatically in the last few years, with the multiparametric magnetic resonance (mpMRI) playing a starring role with the introduction of the “MRI Pathway”. In this scenario the basic tenet of network medicine (NM) that sees the disease as perturbation of a network of interconnected molecules and pathways, seems to fit perfectly with the challenges that PCa early detection must face to advance towards a more reliable technique. Integration of tests on body fluids, tissue samples, grading/staging classification, physiological parameters, MR multiparametric imaging and molecular profiling technologies must be integrated in a broader vision of “disease” and its complexity with a focus on early signs. PCa screening research can greatly benefit from NM vision since it provides a sound interpretation of data and a common language, facilitating exchange of ideas between clinicians and data analysts for exploring new research pathways in a rational, highly reliable, and reproducible way.

## Introduction

Prostate cancer (PCa) is the second common cancer in men worldwide^1^. In their lifetime, 1/6 of men will eventually be diagnosed with prostate cancer, with the prevalence increasing with advancing age^2^. It represents a major health concern, especially in western countries, with their greater proportion of elderly in the general population^3^. PCa is universally acknowledged as a complex disease, given its multi-factorial etiology involving multiple genetic and environmental factors. Consequently, there is an urgent need to resort to an integrated approach mainly focused on the tight interconnections among the many factors involved, from the molecular to the environmental level. “Network medicine” (NM)^4^ is a new approach to complex disease that offers a promise in this regard. What is NM?

A “network” is a collection of point (nodes) that are joined in pairs by lines (edges). This simple definition provides a powerful graphical approach to visualize and analyze relationships between heterogeneous variables of interest. NM is a new field combining principles and approaches from systems biology and network science to understand the causes of human diseases by integrating different sources of clinical and molecular data^4^. Moreover, the network representation of the links among biological entities allows an “explorative analysis” of data using cognitive metaphors such as the topological concepts of “hub” or “community” taken from the social sciences and amenable of biological interpretation^5^. As an illustrative example, increasingly sophisticated network-based approaches have been recently developed for the identification of disease genes and disease pathways, which may also lead to more accurate integrated biomarkers for pathology early detection to monitor the functional integrity of networks that are perturbed by diseases.

Liquid biopsy is an emerging noninvasive diagnostic tool that can target different cancer biomarker (e.g., circulating miRNA, DNA, exosome and cancer cells), and has proved to be a promising diagnostic strategy for breast, prostate, colorectal, and non small cell lung cancer. Approved and commercialized liquid biopsy tests applied to prostate cancer for diagnostic purposes and not for screening are: the ExoDx Prostate (IntelliScore), the Progensa PCA3 and the SelectMDx. Tests on body fluids might provide access to multiple layers of tumor-specific biological information (genomes, epigenomes, transcriptomes, proteomes, metabolomes, circulating tumor cells, and exosomes), and can be integrated, with a network-based method, to the many emerging omics technologies for diverse purposes, among which, we notably find disease early diagnosis^6^.

An effort to go toward precision medicine in oncology, integrating phenotype and genotype to establish standards for translation of the research in cancer biology has been made and described by Halu et al. who build and analyze the multiplex network of 779 human diseases, for both a genotype-based layer and a phenotype-based layer, proposing new disease associations generating a unique feature of the information flow within and across the two layers^7^. Sonawane et al. recently reviewed all the existing network types and biomedical data sources that helped in the identification of driver somatic mutations, the molecular basis of cancer progression, and potential therapeutic interventions for cancer subtypes^8^. Applying such techniques on pre-symptomatic population’s wide screening for early cancer detection is vital possibly improving patients’ clinical outcomes.

Screening for prostate cancer early detection is one the most important topics for its management. Among researchers in the field, there is a growing awareness of the complexity of the task, which may involve the integration of many different data, techniques, and approaches. In our opinion, the new paradigm of NM possess all the features needed to become a key player in the field, given its specific ability to deal with the essential interconnectivity of all the factors and pathways involved in PCa development and, as such, a good candidate to explore complex computational and clinical biomarkers for screening.

## Screening for PCa: a controversial issue

Screening for PCa is one of the most debated topic in urological literature^9^. Up to date, early detection is primarily based on Prostate Specific Antigen (PSA) value tests. Currently, there is strong advice against population-based systematic screening in all countries. International guidelines recommend PSA screening to high-risk individuals with a life expectancy of at least fifteen years^10,11^, benefits exist in terms of lower stage and grade cancer at diagnosis, with still any benefit in terms of mortality in case of aggressive disease^12,13^. However, the discussion on overdiagnosis and overtreatment continues, especially in asymptomatic patients with comorbidities, which might cause considerable harm due to a limited life expectancy^14^. Among the drawbacks, we find unnecessary biopsies due to false-positive PSA tests, overdiagnosis and follow-up of indolent disease, and potential complications from prostate cancer treatment. Also, the potential role of the Digital Rectal Examination (DRE) as an alternative test for early diagnosis has been proved only in patients with a high level of PSA^15^.

Additional information might be gained using alternative tests performed in patients with very high suspicion of PCa with a prior negative biopsy. Among these, we find Progensa-PCA3 and SelectMDX DRE urine tests, the serum 4Kscore and PHI tests or a tissue-based epigenetic test (ConfirmMDx). A small percentage of men with PCa have a real inheritable disease (5-10%) and certain recognized single-point mutations have been linked to an increased risk of PCa such as breast cancer genes 1 and 2 (BRCA1 and BRCA2, respectively), mutL homolog 1 (MLH1), mutS homologs 2 and 6 (MSH2 and MSH6, respectively), postmeiotic segregation increased 2 (PMS2), homeobox B13 (HOXB13), checkpoint kinase 2 (CHEK2), nibrin (NBN), BRCA1-interacting protein C-terminal helicase 1 (BRIP1), and ataxia telangiectasia mutated (ATM)^16^. Instead, in primary prostate cancer, one of seven subtypes defined by specific gene fusions (ERG, ETV1/4, FLI1) or mutations (SPOP, FOXA1, IDH1) have been recognized and presented by The Cancer Genome Atlas (TCGA). They investigated a total of 333 primary prostate carcinomas and they found that 74% of these showed specific gene alterations and defined certain epigenetic profiles that could be actionable in the future with genetic/molecular testing panels^17^. However, no recommendations exist on the use of these tools.

Prostate cancer has very different therapeutic approaches, varying according to its grading and staging classification. Prostate cancer is graded using the Gleason system, which is recommended as an international standard in prostate cancer grading. Gleason score stratifies prostate cancer into five grades of glandular patterns of differentiation. A new grading system for prostate cancer was proposed in 2014 by the International Society of Urological Pathology (ISUP) that introduced the definition of five different Grade Group with different prognostic significance^18,19^. PCa exists as two separate entities with their own natural history. Clinically insignificant prostate cancer (CiPCa) is defined as T1c or T2a, PSA < 10 ng/ml, PSAD < 0.15 ng/ml,< 3 positive cores with <50% cancer, GS 6 (GrG1); 3 + 4, if MR-targeted biopsy performed^20^. Clinically significant cancer (CsPCa) is defined as Gleason score >7, tumor volume >0.5 ml, and/or extra prostatic extension^10^.

Imaging plays a valuable role in the noninvasive detection, localization, grading, and pretreatment staging of prostate carcinoma, being able to differentiate clinically localized disease (e.g., stage T1 or T2), generally amenable to local therapy, from a more advanced disease that may require multimodal therapy. In addition, it plays an important role to carry out biopsies for histopathologic analysis of the tumor^21^. Particularly, multiparametric MRI has become a powerful tool to achieve these goals. Instead, Transrectal ultrasound (TRUS) has proved to be unreliable for PCa detection. MpMRI is increasingly performed in patients with suspect PCa, based on clinical and laboratory data, and it is recommended as first-line study in naive patients, according to the European Association of Urology (EAU) guidelines on PCa and the updated PI-RADS v2.1 recommendations, followed by systematic plus targeted biopsy in case of PI-RADS≥ 3 lesion detection^10,11,22^.

MpMRI and its “pathway” has been extensively studied and validated in recent years as “state of the art” management tool^23–25^ and it has been proved to be a cost-effective exam for prostate cancer detection^26,27^. The Precision clinical trial^28^, the MRI First, the 4 M validating pairing studies^29,30^, and the Cochrane 2019 meta-analysis^31^ have shown the superiority of performing mpMRI followed by targeted biopsy in naïve patient with PCa suspicion, rather than performing systematic biopsy in patients with increased PSA values. MpMRI has significantly increased the accuracy to detect cancer with a Positive Predictive Value (PPV) of 38% (36–40%)^31,32^. MRI detects index lesion in 90% of cases, however in few cases, it misses specific types of lesions, mostly ciPCa, non-index csPCa <1 cm, anterior tumors <0.5 ml and tumors showing the cribriform patter on histopathology^33–42^. MpMRI negative predictive value (NPV) for ISUP G ≥ 2 prostate cancer is 91% (86–94%)^31,38,43^, it varies according to PCa prevalence among countries and upon different definitions of clinically significant PCa. It has been suggested that this wide variability relies on centers’ expertise and excellence, which often differ due to lack of general quality standards^44^. In fact, predictive factors of prostate mpMRI performance relies upon quality control, radiologists’ expertise and the application of clinical and laboratory data in the diagnostic work-up of PCa. Inadequate equipment, protocol optimization, sequence parameters with an altered signal to noise ratio, patients’ preparation and radiologists’ expertise are major reasons of low negative and positive predictive values^45^.

Last, but not least, we believe that Machine Learning (ML) techniques, that are gaining increasing popularity for medical imaging diagnostic applications, can greatly benefit from NM methodologies, also in the prostate cancer diagnostics^46,47^. In precision medicine, it is required to find groups of patients with “similar” genomic profile so that each group can be treated by specific molecular targeting. However, it is well known, that there are cases in which results provided by a ML approach are not reproducible, that is, different groups are found using data from a different cohort of patients^48^. The key point is that ML results are generally prone to over-fitting with noisy data and often not interpretable in clear biological terms, so that the “data pattern” identified may not mechanistically linked to a molecular machinery. In fact, for ML, “data are just data” and the pattern found may not necessarily be consistent with biological constraints. By contrast, NM is inherently explanatory, since it provides a link with an underlying “disease module” in the PPI, which can be a guide for the physician to understand the functional commonalities of the identified group, thus providing a sound biological background rationale for the identified data pattern.

Finally, several drawbacks lie beneath the surface of PCa screening: a generally approved and appropriate tool is still missing. Network medicine might fit in this context to link different and significant variables such as clinical data, genetic profile and diagnostic imaging with MRI and ML techniques, to establish the foundation of PCa screening.

**Fig. 1:**
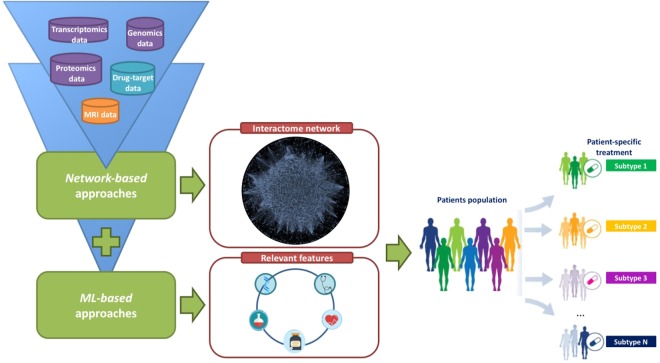
Schematic representation of the integrated computational approach for studying prostate cancer. The cylindrical shapes represent different data types given as input of the computational model: drug-target therapeutic associations data (drug-target data), genomic, transcriptomics, and proteomics data, and Magnetic Resonance Imaging (MRI) data. The rectangular shapes represent the innovative parts that will be ad-hoc developed: (i) network-based approaches to identify disease genes and disease pathways; and (ii) machine learning (ML) approaches to identify the more relevant morphological features related to the pathology, which may help to avoid overdiagnosis and overtreatment. These approaches should be combined for therapy best tailoring.

Data obtained from patients’ follow-up (minimum of 48 months) with PSA measurements, MRI exams (given the high MRI negative predictive value) with application of ML techniques and targeted biopsy results (areas not harboring prostate cancer), integrated to information obtained from body fluid examinations, gene profile and molecular data might be exploited through the application of a network-based method, to obtain meaningful data not solely on cancer cell, rather on normal tissue in order to perform tumor screening and early diagnosis. Primary end-point being a reduction of patients undergoing useless diagnostic exams such as systematic prostate biopsy, which would lead to overdiagnosis and overtreatment (Fig. 1). As secondary end-point, network-based method might offer significant insight on cancer cells development, tumor grading, treatment strategy, and prognosis.

## Network-based approaches in prostate cancer research

NM assumes that all cellular components that belong to the same topological, functional or disease module have a high likelihood of being involved in the same disease. Network-based tools have been applied to a wide range of diseases and pathophenotypes^49,50^, such as several different types of cancer, including prostate cancer (Table 1).

**Table 1:** Summary of network-based approaches to analyze different cancer types, including prostate cancer.

Method	Network type	Database	Cases of study	Data type	Reference
Mode-of-action by network identification (MNI) algorithm	Gene regulatory network	Microarray data from: GEO, Oncomine, EBI ArrayExpress (MEXP-441), Broad Institute Cancer and the St Jude Research	Non-recurrent primary and metastatic prostate cancer	Transcriptomics data	^51^
Drug repurposing based on human functional linkage network (FLN)	Drug-disease perturbed genes network	(1) TCGA: prostate cancer transcriptomics data, (2) OMIM: prostate mutated genes, (3) LINCS: prostate cancer cell line expression in response to more than 4000 drugs, (4) DrugBank: drug data	Prostate cancer, breast cancer, and leukemia	Transcriptomics, Genomics, Drug-target data	^52^
Drug repurposing based on Prostate cancer-specific genome-scale metabolic models (GEMs)	Drug-gene association network	(1) TCGA: prostate cancer transcriptomics data, (2) the Human Protein Atlas: proteome tissue proteome, (3) the Human Pathology Atlas: prostate cancer GEMs, (4) Human Metabolic Atlas: healthy prostate tissue GEMs, (5) ConnectivityMap2: gene expression data from drug-perturbed cancer cell lines	Prostate cancer	Metabolics, Proteomics, Transcriptomics, Drug-target data	^53^
Bayesian network-based approach (Person correlation, mutual information, Kullback Liebler)	Features association network (DAG)	Prostate MR Image Database	Prostate cancer	MR imaging data	^54^
Patients stratification based on network propagation (PRINCE algorithm) and clustering	Protein–Protein interaction network	(1) TCGA: ovarian, uterine, and lung adenocarcinoma somatic mutations data, (2) STRING: protein–protein interactions, (3) HumanNet: protein–protein interactions, (4) PathwayCommons: protein–protein interactions and functional gene interactions	Ovarian, uterine, and lung cancer	Genomics data, Protein–Protein interactions	^56^
Patients stratification based on network propagation (random walk with restart algorithm) and clustering	Protein–Protein interaction network	(1) TCGA: prostate cancer somatic mutations data, (2) STRING: protein–protein interactions TCGA: prostate cancer somatic mutations data	Prostate cancer	Genomics data, Protein–Protein interactions	^55^

Ergün et al. proposed a reverse-engineered gene regulatory network combined with expression profiles to compute the likelihood that genes and associated pathways are mediators of prostate cancer^51^. The algorithm, called mode-of-action by network identification (MNI), is used to infer a model of regulatory interactions between genes. The model is inferred from the training set of microarray expression obtained from a variety of cancer cell lines and it relates changes in gene transcript concentrations to each other. Then, the trained regulatory network is used as a filter to determine the genes affected by a test condition, by computing a z-score designed to boost the likelihood of including genes with significant changes in the test expression profile. By applying this network-based approach to non-recurrent primary and metastatic prostate cancer data, the authors identify the androgen receptor gene (AR) among the top genetic mediators and the AR pathway as a highly enriched pathway for metastatic prostate cancer. The main advantage of this reverse engineering method is its unbiased approach to network mapping, since it is not necessary a priori knowledge of regulatory relationships. However, a bottleneck can be characterized by applying the algorithm to larger and much more sophisticated regulatory networks. In addition, since the inferred connections can only be binary described as “active” or “inhibited”, more complex network relationships involving interactions beyond transcriptional regulation could not be considered.

Network-based methods could also aid drug discovery by exploiting shared similarities among drugs or diseases and infer similar therapeutic applications or drugs selection (drug repurposing). Drug repurposing involves the investigation of existing drugs for new therapeutic purposes and it appears as a promising strategy to identify non-cancer drugs that have anti-cancer activity, as well as tolerable adverse effects for human health. Among network-based approaches for repurposing new therapeutic agents for prostate cancer, Chen et al. exploited the human functional linkage network (FLN) and integrated genomics and drug-response expression data^52^, while Turanli et al. exploited the genome-scale metabolic models (GEMs) and integrated tissue-specific metabolic, proteomics and transcriptomics data^53^.

Chen et al. mapped the prostate mutated genes on the FLN (i.e., an evidence-based weighted network that provides a quantitative measure of functional association among any set of human genes) and select those ones that share a strong functional relationship, whose expression is highly perturbed by the disease, and that are within significantly perturbed pathways of diseases, thus defining the “disease perturbed genes”. Then, they integrated the information about the effects of several drugs on the selected genes expression, thus identifying the drug-response genes. Finally, the authors built two correlation networks composed of drug and disease perturbed genes, defined by genes that are upregulated (downregulated) by the disease and downregulated (upregulated) by the drugs. The greater the drug and disease genes sets correlation, the higher the likelihood that the drug is a viable candidate for repurposing^52^. One of the advantages of this approach is to account for multiple data sources, including genomics and transcriptomics data, functional connectivity and proximity of within module genes. However, as gene expression-based method, it must face the difficulty in defining a robust gene signature due to the existence of noise in gene expression data. In addition, genes used as drug targets and/or regulated by a target may not always show significant expression changes.

Turanli et al. instead exploited a prostate cancer-specific GEMs to build a drug-gene association network composed of candidate drugs and their interacting genes that induce reversal effects in prostate cancer expression for tumor versus non-tumor tissues, and then tested their effect on in vitro cell models^53^. The main advantage of this approach is to integrate transcriptomics proteomics and metabolic data to build a prostate cancer-specific model, facing the issue of inter-tumor heterogeneity (between tumors heterogeneity); however, by constructing a “consensus” model for prostate cancer, the authors did not take into account the intra-tumor heterogeneity (within tumors heterogeneity), which is a relevant point in neoplastic diseases, such as prostate cancer, showing different molecular subtypes. Thus, patients’ stratification could be the most appropriate strategy to find highly efficient drugs for a given subgroup of patients that might have a lower effect in different subgroups.

Conversely, Hussain et al. proposed a network-based approach to study Magnetic Resonance (MR) imaging data^54^. The authors extracted several morphological features from prostate cancer images available from the Prostate MR Image Database and exploited a Bayesian network-based approach to quantify the association between these features. They built a Bayesian network, which is a Direct Acyclic Graph (DAG), whose nodes are ten morphological features and edges represent probabilistic relationships among these variables quantified by using Pearson’s correlation, mutual Information, and Kullback Liebler distance.

Network-based approaches have been also successfully exploited to stratified cancer cohorts into clinically and biologically meaningful subtypes for several cancer types with marked heterogeneity, including ovarian, uterine, lung, and prostate cancer^55,56^. In particular, Hofree et al.^56^ proposed a network-based stratification method, which integrates genome-scale somatic mutation profiles with protein–protein interaction (PPI) network, for ovarian cancer, uterine cancer, and lung adenocarcinoma cohorts. For each tissue, by exploiting a network-propagation algorithm and by clustering together patients with mutations in similar network regions, they obtained a robust patients division into molecular subtypes that were predictive of clinical outcomes, such as tumor histology, overall patient survival, and therapy response. Specifically, they identified three uterine cancer subtypes that were closely associated with the known histological-based subtypes and with the tumor grade; four ovarian cancer subtypes that were significant predictors of patient survival time and of an independent survival with respect to other clinical covariates (e.g., tumor stage, age, mutation rate); and finally six lung cancer subtypes that were also significant predictors of patient survival. Similarly, Yang et al.^55^ integrated somatic mutation profiles in a PPI network for stratifying prostate adenocarcinoma samples. In particular, they mapped prostate cancer mutation profiles into the PPI network and applied a random walk with restart algorithm to spread the effect of each mutation over its network neighborhood, thus obtaining “network-smoothed” patient profiles. Finally, they exploited an unsupervised clustering method based on graph-regularized non negative Matrix factorization to classify the network-smoothed profiles into different molecular subtypes. In particular, they identified three robust molecular subtypes of prostate adenocarcinoma that were associated with most of the clinical and pathological characteristics, such as Gleason score, PSA level, lymph nodes, pathologic N and T stages.

## Emerging opportunities from network medicine for PCa early detection

Recent results using network-based algorithms, as discussed in the dedicated section for PCa, represent just the beginning of a new era of network-based medicine, as witnessed by the plethora of papers on this subject, estimated to be about 3300 from the inception of NM in 2007^57^. The basic tenet of NM, i.e., disease as a perturbation of a network of interconnected molecules and pathways, seems to fit perfectly with the challenges that PCa early detection has to face to advance towards a more reliable—possibly noninvasive—technique. As a matter of fact, tests on body fluids, tissue samples, grading/staging classification, physiological parameters, multiparametric MRI, and many other molecular profiling technologies must be integrated in a broader vision of “disease” and its complexity, with a focus on early signs. In this regard, the field can benefit from the framework and algorithms of NM. Moreover, screening is particularly important for PCa, since its early detection and treatment provide the greatest chance of cure. However, so far, performance relies upon human-based skills that vary considerably among dedicated health centers.

In conclusion, we envisage that PCa screening research can greatly benefit from NM vision and algorithms since it provides a sound biological interpretation of data and a common language (a metaphor) that facilitate the exchange of ideas between clinicians and data analysts for exploring new research pathways in a rational, highly reliable and reproducible way.

## Supplementary information

Reporting Sum
